# Synergistic effects of rare variants of ARHGAP31 and FBLN1 *in vitro* in terminal transverse limb defects

**DOI:** 10.3389/fgene.2022.946854

**Published:** 2022-09-13

**Authors:** Hong Tian, Fan Chu, Yingjie Li, Mengmeng Xu, Wenjiao Li, Chuanzhou Li

**Affiliations:** ^1^ Department of Medical Genetics, School of Basic Medicine, Tongji Medical College, Huazhong University of Science and Technology, Wuhan, China; ^2^ Department of Clinical Laboratory, Affiliated Cancer Hospital of Zhengzhou University, Zhengzhou, Henan, China

**Keywords:** *ARHGAP31*, *FBLN1*, AOS, Cdc42, syndactyly, Limb defects

## Abstract

**Background:** Aplasia cutis congenita (ACC) and terminal transverse limb defects (TTLDs) are the most common features of Adams-Oliver syndrome (AOS). *ARHGAP31* is one of the causative genes for autosomal dominant forms of AOS, meanwhile its variants may only cause isolated TTLD. Here, we report a proband presented with apparent TTLD but not ACC.

**Methods:** Whole exome sequencing (WES) and Sanger sequencing were applied to identify causative genes. Expression vectors were constructed for transfections in mammalian cell cultures followed by biochemical and functional analysis including immunoblotting, immunofluorescence staining, and cell counting kit-8 assay.

**Results:** WES and Sanger sequencing suggested that the proband inherited rare *ARHGAP31* variant [c.2623G > A (*p*.Glu875Lys)] and a rare *FBLN1* variant [c.1649G > A (*p*.Arg550His)] from one of her asymptomatic parents, respectively. Given *FBLN1* variation has also been linked to syndactyly, we suspected that the two genes together contributed to the TTLD phenotype and explored their possible roles *in vitro*. Mutant FBLN1 showed reduced expression resulted from impaired protein stability, whereas ARHGAP31 protein expression was unaltered by mutation. Functional assays showed that only in the co-transfected group of two mutants cell viability was decreased, cell proliferation was impaired, and apoptosis was activated. Cdc42 activity was declined by both ARHGAP31 mutation and FBLN1 mutation alone, and the two together. Furthermore, the MAPK/ERK pathway was only activated by two mutants co-transfected group compared with two wild-type transfections.

**Conclusion:** We report a case carrying two rare variants of limb defects associated genes, *ARHGAP31* and *FBLN1*, and provide *in vitro* evidence that synergistic disruption of cellular functions attributed by the two mutants may potentiate the penetrance of clinical manifestations, expanding our knowledge of clinical complexity of causal gene interactions in TTLD and other genetic disorders.

## Introduction

Adams-Oliver syndrome [AOS (MIM#100300)], also known as AOS1, is a rare inherited disease characterized by aplasia cutis congenita (ACC) and terminal transverse limb defects (TTLDs) as the most common clinical features (Kuster et al., 1988). ACC is mostly present on the scalp, whereas some patients show extra skull defects in various degrees (Adams and Oliver, 1945). TTLD could present different degrees of injury at one limb end to four limbs end. In addition to ACC and TTLD, other common anomalies include central nervous system anomalies, cutis marmorata telangiectasia congenita, and congenital heart defects (Hassed et al., 2017). AOS patients may show severe-to-mild symptoms, but carriers with same gene variant within one family may show phenotypes ranging from no obvious clinical manifestations to severe multi-system abnormalities (Hassed et al., 2017). Clinically, in cases with a known family history, presence of either ACC or TTLD has been considered sufficient to warrant the diagnosis of AOS (Snape et al., 2009).

*ARHGAP31* is one of the causal genes identified in AOS (Hassed et al., 2017; Meester et al., 2018). Previously, Southgate has found two *ARHGAP31* variants in two independent families with patients present with ACC and TTLD, which are the most common clinical features of AOS (Southgate et al., 2011). Later in 2014, an *ARHGAP31* variant in a big family was reported by Isrie et al. (2014), with the only clinical feature of isolated TTLD, suggesting the possibility of segregated phenotype caused by a single gene variant. Along with variants in other causative genes such as *DLL4* and *NOTCH1*, *ARHGAP31* variants have consistently been confirmed in a large cohort of AOS by Meester et al. (2018). As a GTPase-activating protein (GAP) encoded by *ARHGAP31* gene, ARHGAP31 inactivates the Rho GTPases family members Cdc42/Rac1, which master central roles in cell division, survival and migration, and the maintenance of the actin cytoskeleton (Melendez et al., 2011). Truncated forms of ARHGAP31 disrupt the actin cytoskeleton structures through inactivation of Cdc42/Rac1 (Southgate et al., 2011).

Fibulin-1, encoded by *FBLN1*, is an extracellular matrix protein that has an important role in the structure of elastic fibers and basement membranes of various tissues (De Vega et al., 2009). A missense mutation in fibulin-1 in a consanguineous family showed a novel syndrome of syndactyly and other abnormalities in the central nervous system (CNS) (Bohlega et al., 2014), indicative of the crucial role of fibulin-1 in development of the CNS and various connective tissues. Nonetheless, no involvement of FBLN1 in AOS has been reported so far.

Here, we report a proband with similar condition to the cases of arrested development described by Isrie et al. (2014). Using whole exome sequencing and Sanger sequencing, two rare variations were identified. The proband carries an *ARHGAP31* variant (c.2623G > A (p.Glu875Lys) concomitant with *FBLN1* variant (c.1649G > A (*p*.Arg550His). Each of her parents carrying one of the former mutants was phenotypically normal. Since aforementioned *FBLN1* variation has also been shown to be linked with syndactyly (Debeer et al., 2002), we therefore suspected that the two genes together contributed to the TTLD phenotype and explored their possible roles *in vitro*. With exogenous expressions of ARHGAP31 and FBLN1 in mammalian cells, cell viability, target gene expression, cytotoxicity, apoptosis, and related signaling molecules were analyzed, and all the evidence indicate a pro-apoptotic role of FBLN1 in exacerbating the dysfunction of ARHGAP31 caused by variation, ultimately triggering the synergistic effects of penetrance of TTLD phenotype by *ARHGAP31* and *FBLN1* variations.

## Materials and methods

### Patient information

A 30-year-old female was inspected in our consultation center with abnormal finger and toe development, and her parents had no AOS family history of the defects. She is the only child in the family whose mother naturally conceived and gave birth with normal birth parameters. The left hand fingers and two foot toes with abnormal development were observed at birth. There were no other abnormalities during development. Her parents have no history of teratogenic contact. She came for preconception counseling. This study was approved by the Ethics Committee of Tongji Medical College, Huazhong University of Science and Technology. All subjects have signed an informed consent form, and phenotypic photos have been approved for publication in this study.

### Whole exome sequencing and bioinformatics analysis

Peripheral blood from all subjects within the family was collected for genomic DNA extraction. WES of genomic DNA of the proband was performed by Shenzhen BGI Clinical Inspection Center Co., Ltd. Briefly, genomic DNA was first extracted for library preparation. A BGIV4 chip was used to capture and enrich gene exons and DNA adjacent to the clipping region. The MGISEQ-2000 sequencing platform was employed for variants detection and analysis. The quality control index of sequencing data is: the average sequencing depth of target area is ≥180×, and the proportion of sites with average depth of target area >20× is >95%.

Sequenced fragments were compared with UCSC hg19 human reference genome by the Burrows–Wheeler alignment (BWA) to eliminate duplication. Database including the 1000 genomes, ExAC, ESP6500, gnomAD, TOPMed, and BGI local database were referenced. Variants with a frequency of less than or equal to 1% of known causative genes in OMIM was listed in the clinical test report. Genome Analysis Toolkit (GATK) software was used for SNV, INDEL, and genotype detection. ExomeDepth was used to detect at exon level copy number variation. The naming of variation follows the naming standard of Human Genome Variation Society (HGVS). Variant pathogenicity annotation is based on the American College of Medical Genetics and Genomics (ACMG) and the American Society for Molecular Pathology (AMP) sequence interpretation guidelines, and with reference to interpretation of the guidelines from the ClinGen Sequence Variant Interpretation working group and the British Society for Clinical Genome Sciences (ACGSs).

### Validation of variations *via* Sanger sequencing

*ARHGAP31* and *FBLN1* variants were identified by aforementioned WES. Variations in *ARHGAP31* and *FBLN1* genes were validated by Sanger sequencing. The PCR products were sequenced on an ABI 3130 DNA Analyzer (Applied Biosystems, United States). The sequencing data were visualized in DNA Star software to confirm the variations. We carried out family analysis in order to explore the source of variations in *ARHGAP31* [c.2623G > A (*p*.Glu875Lys)] and *FBLN1* [c.1649G > A (*p*.Arg550His)]. Three DNA samples were extracted respectively from peripheral blood lymphocytes of patient and her parents using a genomic DNA extraction kit (Cwbio Company, CW 2087M). Sanger sequencing were performed using primers as follows: *ARHGAP31* forward primer: 5′-CCA​GGC​AAT​CTG​TCT​CCT​CC-3′, *ARHGAP31* reverse primer: 5′-CAT​GCT​GGG​CAA​TGT​CTG​TC-3′; *FBLN1* forward primer: 5′-CGT​CTC​CAG​ATG​GGT​ATG​GC-3′, and *FBLN1* reverse primer: 5′-CCC​CTT​GAC​TTT​CCG​AGA​CC-3′.

### Construction of *ARHGAP31-* and FBLN1-expressing vectors

The C-terminus of coding sequences (CDS) for *ARHGAP31* (4335 bp) and *FBLN1* (2052 bp) were added with CDS for FLAG (DYKDDDDK) tag and hemagglutinin (HA, YPYDVPDYA) tag, respectively. *ARHGAP31* (c.2623G > A) and *FBLN1* (c.1649G > A) mutations were directly incorporated in the sequence design by substituting the corresponding nucleotide in wild-type sequences. The above full-length sequences with and without mutations were straightforwardly synthesized by TIANYI HUIYUAN biotechnology (Wuhan, China), using synthetic oligonucleotide primers-based PCR amplification. CDS for ARHGAP31-FLAG was then subcloned into eukaryotic-expressing vector pcDNA3.1 using HindIII/NotI restriction sites, while EcoRI/NotI sites were used for cloning of FBLN1-HA into pcDNA3.1. Successful insertions were verified by digestion using corresponding restriction enzymes followed by Sanger sequencing. To induce site-directed mutagenesis at c.2047C > T to construct the *p*. Gln683X mutant form of *ARHGAP31*, PCR amplification of the entire plasmid (pcDNA3.1-ARHGAP31-wild-type-FLAG) was performed using primer pair against c.2047C > T site (*p*. Gln683X-FP: 5′-CCA​ATT​TAG​CCT​ATT​CTC​GAG​TCG-3′; *p*. Gln683X-RP: 5′-TAG​GCT​AAA​TTG​GGC​TGG​TCT​TCA-3′). PCR product was digested with DPN1 enzyme to eliminate original plasmid template before transformed into competent E. coli cells. Clones were sent for Sanger sequencing to confirm each single nucleotide including the mutation.

### Cell culture and plasmid transfection

The human embryonic kidney HEK293T cell line and human cervical cancer HeLa cells line were obtained from China Center for Type Culture Collection (CCTCC). The cells were routinely maintained in Dulbecco’s modified eagle medium at 37°C (5% CO_2_) (Gibico). The medium was supplemented with 10% fetal calf serum (Life Technologies) and 1% penicillin–streptomycin (10,000 U/ml, Life Technologies). The cells were cultured in 96-well plates for cell proliferation assays, and in 12-well plates and Petri dishes for immunofluorescence and Western blotting analysis. The cells were plated onto plates for 18–24 h before transfection at a confluence of 60%–70% or onto coverslips as required.

Previously indicated plasmids and control vector (pcDNA3.1) were prepared and quantified before transfections. Prior to transfections, the cells were cultured to 70%–80% confluence. Transfection was conducted using polyethylenimine reagent (PEI, Polyscience). Briefly, for each well in a 12-well/plate, 1 μg DNA was diluted in 200 μl Opti-MEM (Gibco) followed by mixing with 1 μl PEI solution (5 mg/ml). Transfection mixture was incubated at room temperature for 20 min before added into each well. The cells were harvested at 24 h or 48 h post-transfection for immunofluorescence, Western blotting, and Cdc42 activity as indicated.

### Cell counting Kit-8 assay

HeLa cell suspension was inoculated at 5,000 cells per well in a 96-well plate, which was later pre-incubated for 24 h in a humidified incubator at 37°C, 5.0% CO_2_ with saturated humidity. Prepared expression plasmids were transfected using PEI reagent. For each well, 10 μl transfection mixture including 200 ng of total DNA and 0.5 μg PEI was incubated and added into the cell medium; 24 and 48 h after transfection, 10 μl of CCK-8 solution (Biosharp Life Science, BS350A) was added to each well of the plate. Bubbles were avoided, and plates were incubated for 1 h before absorbance was measured at 450 nm with a microplate reader.

### Quantitative real-time PCR

HeLa cells transfected with FBLN1 plasmids were collected in an RNA isolator (Vazyme Biotech, R401) for total RNA extraction, followed by reverse-transcription using a HiScript II 1st strand cDNA synthesis kit (Vazyme Biotech, R212-01). The resulting cDNA was used for qRT-PCR using a SYBR Green Master mix (Vazyme Biotech, Q221-01) and analyzed using the QUANTAGENE q225 real-time PCR machine (KUBO biotechnology). The following primer pairs were used: Primer Pair1 forward, 5′-GGA​TCT​CTC​TCG​CCA​CGG-3′, Primer Pair1 reverse, 5′- TCA​AGC​GTA​TGT​CTG​GGA​C-3′ and Primer Pair2 forward, 5′- CTC​ACC​AAG​CCT​GTC​CCC-3′, Primer Pair2 reverse, 5′- TCA​AGC​GTA​TGT​CTG​GGA​C-3′. *GAPDH* forward, 5′- TGC​ACC​ACC​AAC​TGC​TTA​GC-3′, *GAPDH* reverse, 5′- GGC​ATG​GAC​TGT​GGT​CAT​GAG-3′. The 2-ΔΔCT method has been used for fold change analysis using *GAPDH* as internal control.

### Protein stability analysis

Protein stability was assessed using protein translation inhibitor cycloheximide (CHX, Merck Sigma-Aldrich, C7698) treatment. HeLa cells were transfected with FBLN1 plasmids for 24 h and subjected to CHX treatment at final concentration of 50 μg/ml. The cells were collected and lyzed at indicated time points (0, 2, 4, 6, 8, and 12 h) after treatment, followed by immunoblotting.

### Co-immunoprecipitation

Co-immunoprecipitation was performed following standard procedures. In brief, the cells were washed in PBS and lyzed in cold NP-40 buffer (50 mM Tris-HCl, pH 7.4, 150 mM NaCl, 2 mM EDTA, 1% NP-40) supplemented with a protease/phosphatase inhibitor cocktail (Cell Signaling Technology). The lysate was briefly sonicated and centrifuged at 12,000 rpm for 10 min at 4°C. The supernatant was then collected and 1 mg of total protein lysate was incubated with protein A-conjugated agarose beads (Beyotime technology, P2051) and primary antibodies overnight at 4°C. Anti-FLAG antibody (Beyotime biotechnology, AF519, 1: 250 dilution) and anti-HA antibody (Beyotime biotechnology, AF0039, 1:250 dilution) were used. The beads were spun down, washed with PBS buffer, and denatured with 2× Laemmli sample buffer (Bio-Rad), followed by Western blotting for validation.

### Western blotting

The cells were collected in 1× radioimmunoprecipitation assay (RIPA) buffer (Cell Signaling Technology) supplemented with a protease/phosphatase inhibitor cocktail (Beyotime biotechnology) or 2× Laemmli sample buffer (Bio-Rad). They were then thoroughly lyzed by sonication and heat denatured at 95°C for 5 min before loading onto gels for analysis. Equal amounts of protein were loaded (10–50 μg depending on the assay) and resolved by SDS-PAGE in Tris-glycine-SDS buffer, followed by transfer onto nitrocellular membranes (millipore). The membranes were blocked with blocking buffer (Beyotime P0256) for 0.5 h and then reacted with primary antibodies in primary antibody dilution buffer (Beyotime P0023A) overnight at 4°C under gentle rocking. Primary antibodies are as follows: anti-FLAG antibody (Beyotime biotechnology, AF519-1, 1:2,000 dilution), anti-HA antibody (Beyotime biotechnology, AF0039, 1:2,000 dilution), anti-actin antibody (Proteinab Biotech, Cat#10003-M01, 1:5,000 dilution), anti-GAPDH antibody (EMD Millipore, AB2302, 1:5000 dilution), anti-Cdc42 antibody (Cytoskeleton, Cat#ACD03, 1:250 dilution), anti-pERK1/2 antibody (Cell signaling Technology, #4370, 1:2,000 dilution), and anti-ERK1/2 antibody (Cell signaling Technology, #4696, 1:2,000 dilution). After washing, the corresponding IR Dye 680RD/800CW secondary antibodies (LICOR, 1:10,000 dilution) were added for 1 h at room temperature under constant agitation. After washing with Tris buffered saline wash buffer with Tween 20, the membrane was scanned in the Odyssey Fc Imaging System (LICOR) for detection of an infrared signal. For the Cdc42 activity experiment, the corresponding horseradish peroxidase (HRP)-conjugated anti-rabbit secondary antibodies (Beyotime biotechnology, 1:1000 dilution) was used, and the membrane was probed with enhanced chemiluminescence (ECL) Western blotting substrate (Beyotime biotechnology P0018AM) and visualized using a Tanon scanner system (Tanon5200).

### Assessment of Cdc42 activity

Cdc42 activation assay was examined using the Cdc42 Activation Assay Biochem Kit (Cat. # BK034, cytoskeleton), following the manufacturer’s instructions. HeLa cells were grown at approximately 70%–80% confluent prior to transfection. The cells were treated with HB-EGF (C600219, Sangon Biotech) for 3 min before lyzed in cell lysis buffer. Then, 10 μl PAK-PBD beads were added to equivalent amount of lysate (800 µg total protein) with gentle rotation for 1 h at 4°C. After three washes, the beads were resuspended and processed for immunoblotting using total Cdc42 rabbit monoclonal antibody (Beyotime biotechnology, AF2794, 1:2,000 dilution).

### Cell proliferation using EdU labeling

EdU cell proliferation image kit (green fluorescence) from Abbkine (Cat#. KTA 2030) was used, following manufacturer’s instructions. Briefly, the cells grown on coverslips were transfected with desired plasmids for 24 h and were incubated with 10 μM EdU solution for 2 h. After incubation, the cells were fixed in 4% paraformaldehyde (PFA) for 10 min and were subjected to Click-iT reaction mix including copper reagent, AbFluor 488 azide, and reducing agent for 30 min. The cells were washed and counterstained with DAPI to visualize the nuclei. Fluorescence images were captured at 10× objective by using an inverted fluorescence microscope (ICX41, Sunny optical technology). The images were analyzed with ImageJ.

### Live and dead cell double staining

Live and dead cell double staining kit from Abbkine (Cat#. KTA1001) was used, following manufacturer’s instructions. Briefly, the cells were transfected as desired for 24 h; 1× assay buffer was prepared and warmed. LiveDye and NucleiDye were diluted at 1/1,000 dilution in assay buffer as staining solution. The cells were washed with PBS twice before incubated with staining solution at dark for 30 min. After staining, the cells were washed with PBS again and subjected for imaging immediately. Fluorescence images were captured at 10× objective by using an inverted fluorescence microscope (ICX41, Sunny optical technology). The images were analyzed with ImageJ.

### TUNEL (TdT-mediated dUTP Nick End Labeling) assay

TUNEL apoptosis detection kit was purchased from Yeason Biotechnology (Cat. # 40306ES20). Briefly, the cells grown on coverslips were transfected with desired plasmids for 24 h before fixation at 4% PFA, followed by permeabilization with 0.3% Triton X-100 in PBS. For the positive control, permeabilized cells were with treated DNase I (10 U/ml) solution for 10 min at room temperature and washed with deionized water thoroughly. TdT-labeling mix was prepared with FITC-12-dUTP labeling mix and recombinant TdT enzyme in equilibration buffer. Negative control was performed using labeling mix without TdT enzyme. The cells were washed after labeling at 37°C for 1 h and counterstained with DAPI to visualize the nuclei. Fluorescence images were captured at 10× objective by using an inverted fluorescence microscope (ICX41, Sunny optical technology). The images were analyzed with ImageJ.

### Immunofluorescence staining

Cells grown on coverslips were washed gently with phosphate buffered saline (PBS) and fixed in 4% PFA (except that cells were fixed in cold methanol for endogenous ARHGAP31 staining), followed by permeabilization with 0.3% Triton X-100 in PBS. The cells were then blocked for 1 h in 5% goat serum, followed by incubation overnight with primary antibodies at 4°C. Anti-FLAG (Beyotime biotechnology, AF519, 1:500 dilution), anti-GM130 (Sigma-Aldrich, #610822, 1:200 dilution), anti-ARHGAP31 (CdGAP (G-8), Santa Cruz, sc-393839, 1:25 dilution), anti-HA (Beyotime biotechnology, AF0039, 1:200 dilution), and antibodies were used. Secondary antibodies were Alexa Fluor (488, 555)-labeled goat antimouse and goat antirabbit antibodies (Thermo Fisher, 1:500 dilution). DAPI was used to visualize the nuclei. Fluorescence images were captured with a 20× or 63× objective using a Zeiss LSM780 laser scanning confocal microscope.

### Statistical analysis

Statistical analysis was performed using Prism 9 software (GraphPad). The biochemical analysis was performed using a minimum of three biological replicates per condition, and randomization of experimental groups was not required. The data distribution was assumed to be normal with similar variance between groups; however, datasets were first assessed with normality tests. Two groups were analyzed with the unpaired Student’s t test when the datasets were normally distributed, otherwise non-parametric tests were used with a non-paired Mann–Whitney test. Three or more groups were assessed with one-way ANOVA followed by a post hoc test for multiple comparisons. For statistical analysis of positivity in fluorescent images, Kruskal–Wallis test were used followed by Dunn’s multiple comparisons test. Wild-type groups always serve as controls in multiple comparisons. All comparisons were performed whenever possible with all significant changes being indicated on the graphs unless changes are not significant. Changes close to significant are also presented. Values are presented as the mean ± standard error of mean (s.e.m).

## Results

### Clinical manifestations and identification of variations

**FIGURE 1:**
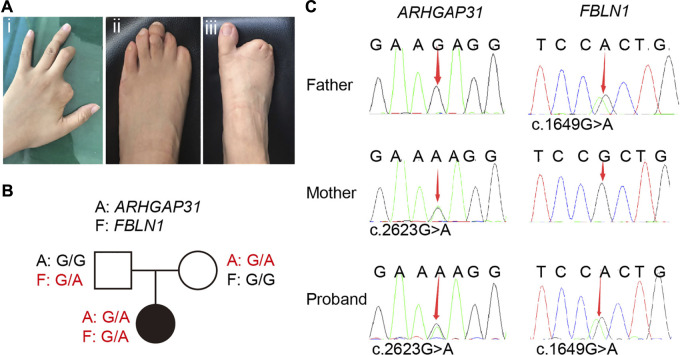
Clinical manifestations in the proband and related sequencing information. **(A)** Defects of the distal digits. (i) Second finger is completely absent, the middle and distal phalanges of the left third finger are missing; (ii) distal limb reduction appeared on the left foot in the first and second left toe with hypoplastic toenails on the third and fifth toes; (iii) her second, third, fourth, and fifth toes of the right foot are cutaneous syndactyly. **(B)** Family pedigree of the proband. **(C)** Sanger sequencing of the family members revealed inheritance of gene variants from her parents.

The proband is a female who has normal intelligence and no obvious phenotypic abnormalities in cardiovascular and nervous systems. As indicated, the middle and distal phalanges of the left middle finger are missing, and the second finger on the left hand is completely absent; distal limb reduction appeared on the left foot in the first and second left toe; her second, third, fourth, and fifth toes in the right foot showed cutaneous syndactyly. In addition, the fourth finger on the left hand and the third toe on the left foot appear to have constriction rings (Figure 1A). No other abnormalities were observed at birth, and there was no other banding on the body elsewhere.

Her parents were assessed by a clinical geneticist and are clinically normal with no family history, we therefore first speculated typical inheritance of two recessive copies on one causative gene, or a *de novo* variation occurred in a pathogenic gene in the proband. Therefore, whole exome sequencing (WES) analysis were first performed in the proband. In general, a total of 25,539.75 Mb of original data were generated, about 98.33% of the target bases were covered with at least 20× per individual, and the mean depth of coverage for all target regions was 201.52. Surprisingly, WES specifically identified two independent variations in two genes, other than classic two copies of variation in one gene. Based on the database and annotation, the allele frequency of *ARHGAP31* (NM_020,754.4): c.2623G > A is absent in gnomAD and is 2/125,568 in TOPMed, while that of FBLN1(NM_006,486.3): c.1649G > A variant is 3/282,786 in gnomAD and is 1/264,690 in TOPMed. Next, the pathogenicity prediction indicates that the *ARHGAP31* variant is deleterious from SIFT (0.03) and benign from PolyPhen (0.211), while the FBLN1 variant is deleterious from SIFT (0.01) and probably damaging from PolyPhen (0.993). We then attempted to assess whether the variations are *de novo* in the family. Sanger sequencing confirmed that the proband carried each copy of the variation from one of the parents (Figure 1B), and the variations in *ARHGAP31* and *FBLN1* cause Glu875 to Lys (E875K) and Arg550 to His (R550H) missense mutations, respectively (Figure 1C). Since *ARHGAP31* is one of the causative genes identified for AOS, and *FBLN1* variation has been shown to cause syndactyly, we highly suspected the two variations together contributes to the TTLD phenotype in the proband.

### Exogenous expression of *ARHGAP31* variant in cultured cells

**FIGURE 2:**
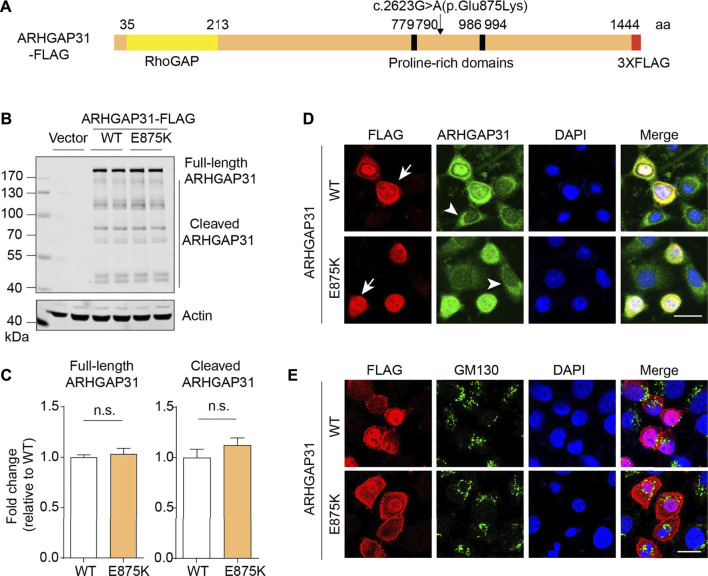
Exogenous expression and localization of ARHGAP31 in mammalian cells. **(A)** Schematic illustration of molecular structure of FLAG-tagged ARHGAP31. Missense mutation site was shown. **(B)** Representative immunoblots of exogenous ARHGAP31 expression in HeLa cells using anti-FLAG antibody. **(C)** Quantification of B (*n* = 4 per group). **(D)** Representative immunofluorescence co-staining of ARHGAP31 expression with anti-FLAG antibody and anti-ARHGAP31 antibody in transfected HeLa cells. Blue, DAPI-labeled nuclei. Arrow denotes ARHGAP31-overexpressing cells and arrowhead denote cells with endogenous ARHGAP31 expression. **(E)** Colocalization of ARHGAP31 and Golgi marker protein GM130 in transfected HeLa cells. Scale bar, 20 μm.

Next, we attempted to investigate gene functions by overexpressing the encoding genes in HEK293T cells. As illustrated in Figure 2A, open reading frame sequences were fused with FLAG-tag. Clinically identified variations and corresponding mutated amino acid were indicated. Protein expression was validated by immunoblotting with anti-FLAG antibodies. Exogenous WT and mutant ARHGAP31 expression showed similar band patterns, with full-length ARHGAP31 at size around 200–250 kDa and a group of weaker cleaved bands below (Figure 2B). Size of full-length ARHGAP31 was validated with antibody against endogenous expression (data not shown). Expression levels were not altered between groups (Figure 2C).

We further looked into the cellular localization of ARHGAP31 in HeLa cells. Anti-FLAG tag labeling and anti-ARHGAP31 staining indicated an obvious intracellular expression including a strong nuclear expression and diffuse cytosolic expression. Our ectopic ARHGAP31 was successfully detected (Figure 2D). Meanwhile, in both groups, cells bearing higher expression of ARHGAP31 appeared more roundish outline than those with lower expression. No obvious localization change was caused by mutation. Since Golgi-specific staining has been previously reported, we stained the Golgi maker protein GM130, and results show that ARHGAP31 was apparently not, least not exclusively, localized at Golgi department (Figure 2E).

### Defective expression of FBLN1 mutant is caused by impaired protein stability

**FIGURE 3:**
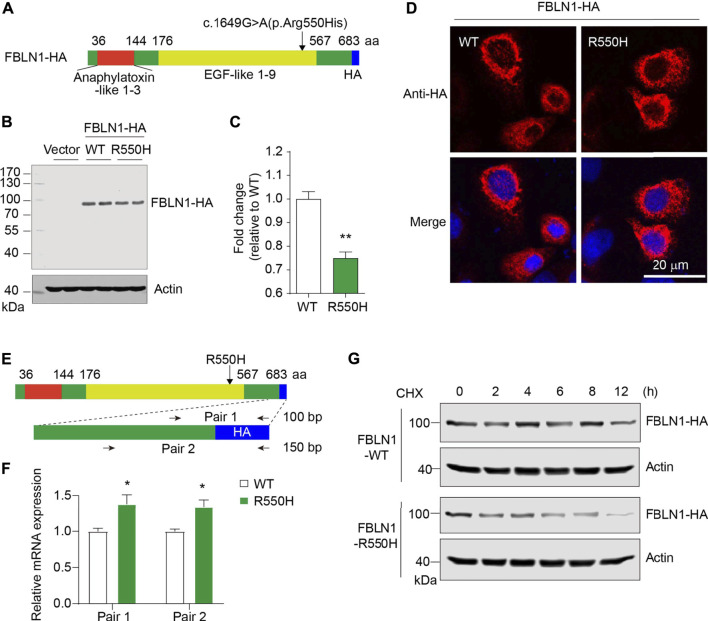
Defective expression of FBLN1 mutant is caused by impaired protein stability. **(A)** Schematic illustration of molecular structure of HA-tagged FBLN1. Missense mutation site was shown. **(B)** Representative immunoblots of exogenous FBLN1 expression in HeLa cells using anti-HA antibody. **(C)** Quantification of B (*n* = 3–4 per group). ** *p* < 0.01. **(D)** Representative immunofluorescence staining of FBLN1-HA in transfected HeLa cells. **(E)** Primer design for quantitative real-time PCR at C-terminus of FBLN1 construct. **(F)** Quantitative real-time PCR results suggesting mutant FBLN1-transfected cells showed more mRNA expression. *n* = 3 per group. *p < 0.05. **(G)** Transfected HeLa cells were treated with protein translation inhibitor cycloheximide (CHX, 50 μg/ml) and subjected to immunoblotting.

Similarly, we constructed FBLN1-HA plasmid as indicated (Figure 3A). Anti-HA immunoblotting revealed an obvious FBLN1 expression at around 90 kDa as predicated. Total protein expression of FBLN1 were significantly reduced by 25% (Figures 3B,C). Anti-HA immunostaining showed that FBLN1 expressed at perinuclear and cytoplasmic departments. A fibrillary-like pattern was observed in both WT and mutant FBLN1-transfected cells (Figure 3D).

To examine the reason for the defective FBLN1 mutant expression, we designed two pair of primers spanning the coding sequence and the HA-encoding region in the construct (Figure 3E). Quantitative real-time PCR revealed that the R550H mutant form of FBLN1 have significantly higher mRNA levels (1.3-fold, Figure 3F). To further dissect the inconsistency, we investigated the protein stability by treating cells with protein translation inhibitor cycloheximide (CHX). As indicated, FBLN1 mutant showed apparently reduced total FBLN1 than the wild-type since 4 h after treatment, and the effect lasted until 12 h (Figure 3G). Hence, the decreased protein stability in the mutant may contribute to the deficiency of the ultimate protein expression.

### Dysregulated protein expression by co-transfection of the variants

**FIGURE 4:**
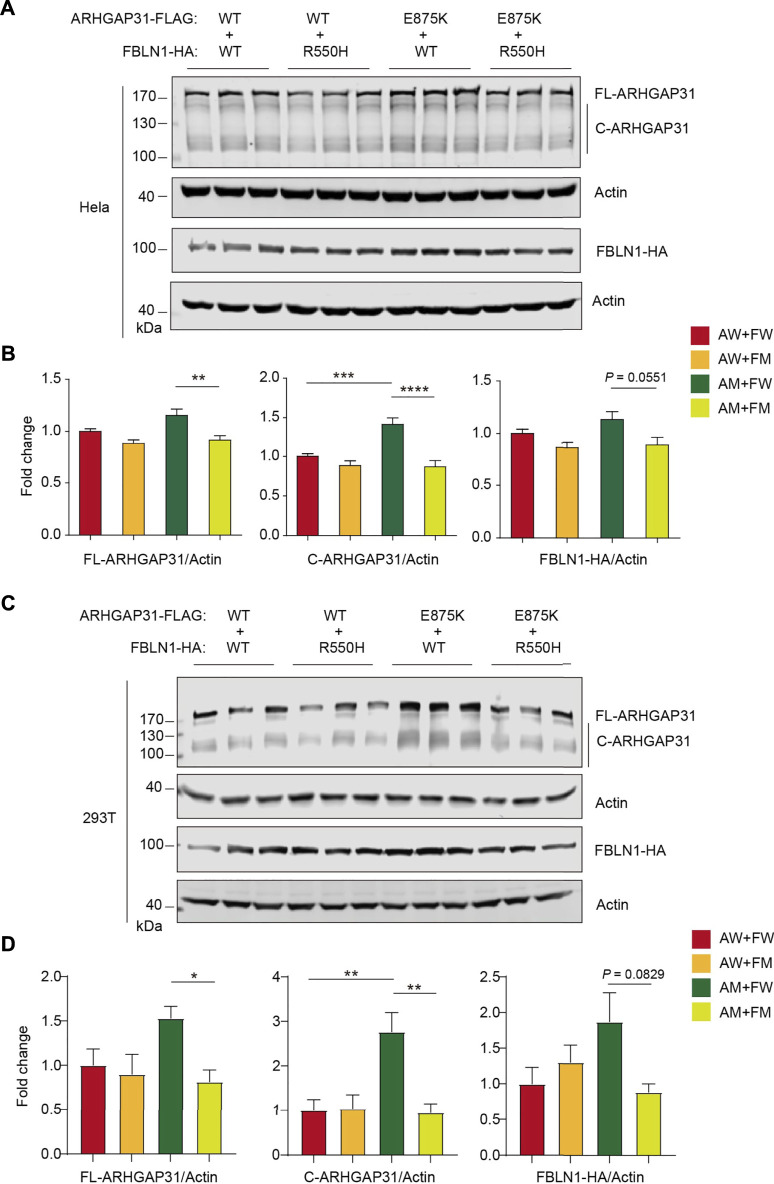
Protein expression with ARHGAP31 and FBLN1 variations *in vitro*. **(A)** Immunoblotting of exogenous ARHGAP31 and FBLN1 in co-transfected HeLa cells as indicated. **(B)** Quantification of **(A)**. **(C)** Immunoblotting of exogenous expression of ARHGAP31 and FBLN1 in co-transfected HEK293T cells. **(D)** Quantification of **(C)**. FL-ARHGAP31, full-length ARHGAP31; C-ARHGAP31, cleaved ARHGAP31; AW, wild-type ARHGAP31; AM, mutant ARHGAP31; FW, wild-type FBLN1; FM, and mutant FBLN1. *p < 0.05, **p < 0.01, ***p < 0.001, and ****p < 0.0001.

To examine possible effects by two mutant proteins together, we performed co-transfection in two types of mammalian cells. In FBLN1-WT-expressing HeLa cells, mutation in ARHGAP31 caused a mild but non-significant increase in full-length protein expression, and a significantly increased cleaved products (*p* < 0.001) (Figures 4A,B). Surprisingly, on the basis of ARHGAP31-E875K expression, all of the full-length, cleaved ARHGAP31 and FBLN1 expressions were significantly compromised in the FBLN1-R550H group compared with the FBLN1-WT group (Figures 4A,B), suggesting possible cytotoxic effects caused by FBLN1 mutant. More interestingly, when the same vector sets were co-transfected into HEK293T cells, we observed extremely similar alternation pattern as we have found in HeLa cells (Figures 4C,D).

### Increased colocalization of *ARHGAP31* and FBLN1 mutants

**FIGURE 5:**
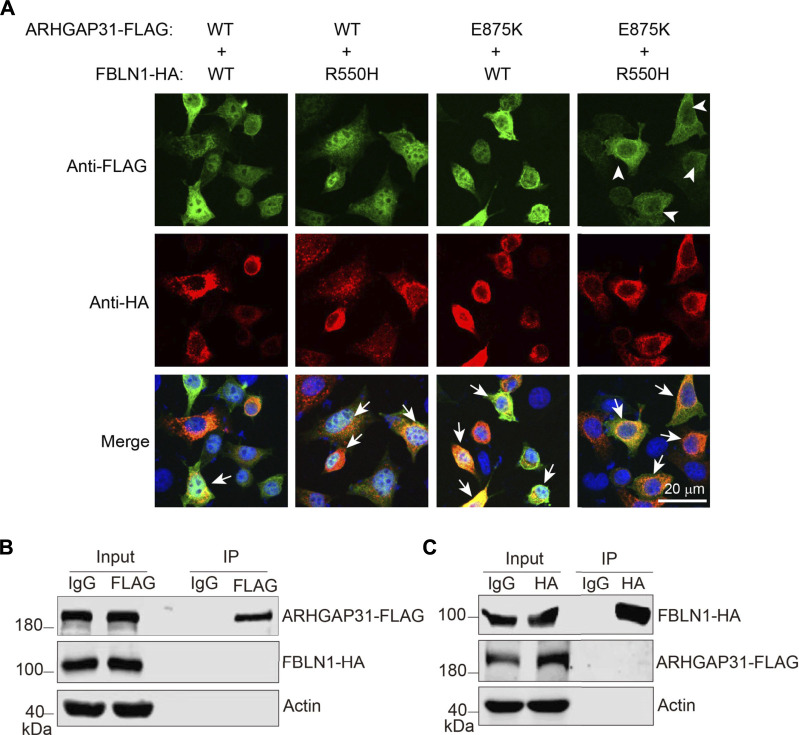
Increased colocalization with mutant ARHGAP31 and FBLN1. **(A)** Representative immunofluorescence staining of exogenous ARHGAP31 and FBLN1 in co-transfected HeLa cells. Green, anti-FLAG staining; red, anti-HA staining; and blue, DAPI-labeled nuclei. Arrowheads indicate cells with apparent non-nuclear expression of ARHGAP31, and arrows indicate recognizable cells with co-localized target proteins. **(B)** HeLa cells were co-transfected with ARHGAP31-FLAG and FBLN1-HA, and co-immunoprecipitation was performed using anti-FLAG antibody followed by detection with anti-HA antibody. **(C)** ARHGAP31-FLAG and FBLN1-HA co-transfected cells were lyzed, and co-immunoprecipitation was performed using anti-HA antibody followed by immunoblotting with anti-FLAG antibody.

We further investigated the subcellular localization of these variants in co-transfected HeLa cells. Compared to the intense nuclear localization of ARHGAP31 when it was transfected alone, apparent non-nuclear expression of ARHGAP31 appeared in both mutants-transfected cells. Overall, FBLN1 localization was not altered between groups. However, co-transfected of ARHGAP31-WT and FBLN1-WT showed only a few cells with relatively balanced expression of both proteins, as indicated by arrows; whereas in either of the mutants, colocalization of ARHGAP31 and FBLN1 were obviously increased (Figure 5A). Furthermore, enhanced co-localization of ARHGAP31 and FBLN1 mutants showed up in the cytosol, suggesting a potential interaction between the two proteins.

To examine if these two proteins directly interact with each other, we overexpressed two proteins in HeLa cells and performed co-immunoprecipitation. Although proteins were successfully immunoprecipitated by anti-tag antibodies, we were not able to detect any obvious interacting target (Figures 5B,C). Therefore, increased co-localization may be due to a direct interaction-independent manner.

### Enhanced cytotoxicity and apoptosis with suppressed cell proliferation triggered by ARHGAP31 and FBLN1 mutants

**FIGURE 6:**
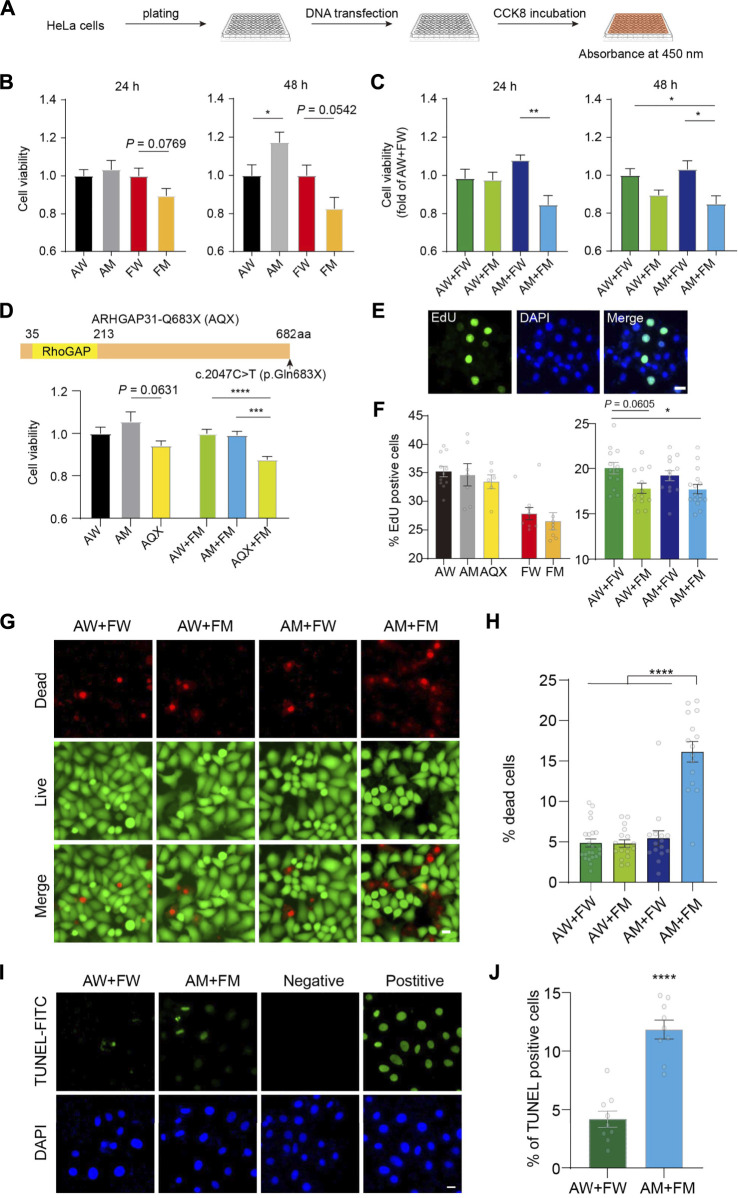
Enhanced cell toxicity and apoptosis with impaired cell proliferation induced by ARHGAP31 and FBLN1 mutants. **(A)** Flowchart demonstration of assessment of cell viability *in vitro* using CCK-8 assay. **(B)** Assessment of cell viability in HeLa cells 24 h and 48 h after single transfection of indicated vectors. **(C)** Assessment of cell viability in HeLa cells 24 h and 48 h after co-transfection of WT and mutant combinations. **(D)** Construction of ARHGAP31-p.Gln683X (AQX) truncation and CCK-8 assay at 24 h after indicated transfections in HeLa cells. **(E)** Representative images showing EdU-positive cells, with DAPI labeling the nuclei. **(F)** Quantification of percentage of EdU-positive cells in transfected HeLa cells as indicated. *n* = 6–13 images including 1,700–5,000 cells per group. **(G)** Representative images showing co-stained live (green) and dead (red) HeLa cells after transfection with quantification in **(H)**. *n* = 15–22 images including 5,000–10,000 cells per group. **(I)** Representative images showing TUNEL-positive HeLa cells after transfection with quantification in **(J)**. *n* = 9 images including 800–900 cells per group. DAPI stains the nuclei. AW, wild-type ARHGAP31; AM, mutant ARHGAP31; FW, wild-type FBLN1; and FM, mutant FBLN1. Scale bar, 20 μm. Individual values are plotted in the bar chart. *p < 0.05, **p < 0.01. ***p < 0.001, and ****p < 0.0001.

To further test the functional consequences of the mutants, we performed CCK-8 assay, which examines combined effects of cell proliferation and cytotoxicity. As illustrated in Figure 6A, CCK-8 assays were conducted 24 h and 48 h after seeding and transfection. An increased cell viability with mutant ARHGAP31 (AM) was observed compared to the WT (AW) at 48 h (*p* < 0.05) but not 24 h, and mutant FBLN1 (FM) showed inhibitory effect trends toward significance on cell activity at both time points (*p* = 0.0769 and 0.0542, respectively, Figure 6B). After 24 h and 48 h with transfection combinations, combination of the mutant genes (AM + FM group) appeared significantly reduced cell viability compared to the AM + WT-FBLN1 (FW) group. In particular, a significant impairment of cell viability was observed in the AM + FM group at 48 h compared to the AW + FW group (Figure 6C). Other comparisons were not statistically significant.

Using the same method, we tested if a previously reported variant *p*. Gln683X of ARHGAP31 (AQX) in AOS exhibits toxicity in our *in vitro* model. Site-directed mutagenesis resulted truncated ARHGAP31 expression, and AQX-transfected HeLa cells showed decreased cell activity compared with AM-transfected cells (*p* = 0.0631) at 24 h post-transfection. More interestingly, when co-transfected with FM plasmid, AQX showed strong toxicity compared with AW and AM (Figure 6D), confirming that AM is less toxic than a known causative variant in AOS.

Given CCK-8 assay indicate a mixed reflection of cell activity, resulting from proliferation and cytotoxicity, we assessed cell proliferation directly using EdU labeling (Figure 6E). Statistical analysis suggested that neither ARHGAP31 nor FBLN1 is not affecting cell proliferation when transfected alone. However, when compared with the AW + FW group, AW + FM showed prone to significant effect in inhibition of cell proliferation; as expected, two mutants together (AM + FM) exhibit significant suppression in cell proliferation (*p* < 0.05, Figure 6F), suggesting a possible role of FBLN1 in regulating cell proliferation.

We next wondered whether cell death is also the cause for the declined cell activity. First, using cell membrane permeability-dependent dye, live and dead cells were stained in four co-transfected groups (Figure 6G). The number of dead cells in the AM + FM group were significantly higher than other groups (*p* < 0.00001, Figure 6H). Next, we directly labeled apoptotic cells using classic TUNEL assay, and the result showed apparently increased cell apoptosis in the AM + FM group compared to the AW + FW group (Figures 6I,J). Together, these results again suggest that ARHGAP31 and FBLN1 mutations may synergistically impair cell activity, which attributes to suppression of cell proliferation and augmented cell death particularly apoptosis.

### Cdc42 inactivation and MAPK/ERK activation by ARHGAP31 and FBLN1 mutants

**FIGURE 7:**
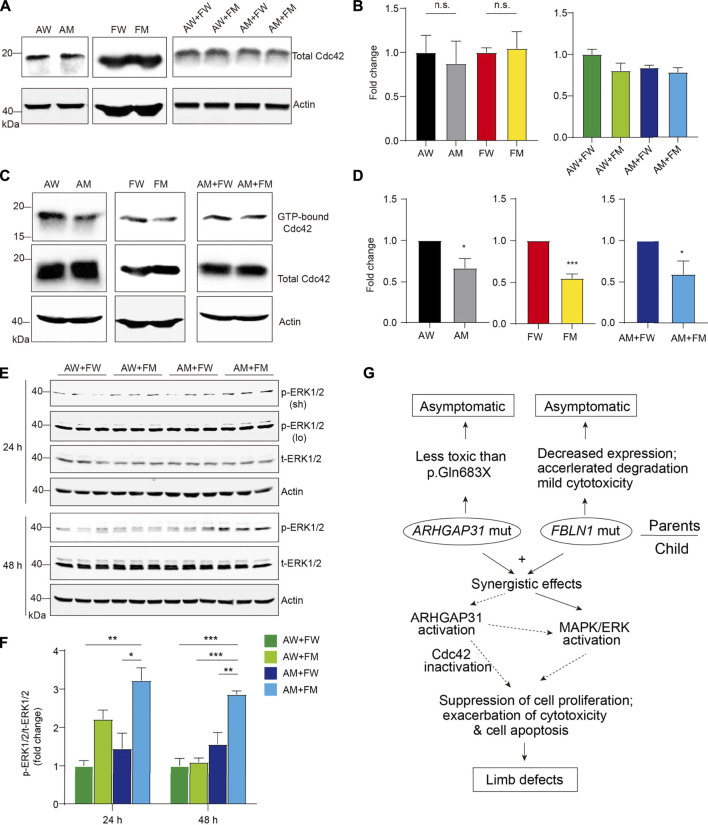
Enhanced activation of Cdc42 by ARHGAP31 and FBLN1 mutants. **(A)** Immunoblotting of Cdc42 expression in HeLa cell lysates after transfection. **(B)** Quantification of A (*n* = 3–6 per group). **(C)** Active Cdc42, that is, GTP-bound form, were detected using specific pull-down assay followed by immunoblotting of total Cdc42 in HeLa cell lysates post indicated transfections. All quantifications of respective densitometry are shown in **(D)** (*n* = 3–4 per group). **(E)** HeLa cells were transfected with indicated plasmids and immunoblotted with pERK1/2 and total-ERK1/2 (t-ERK1/2) antibodies. Sh, short exposure and lo, long exposure. **(F)** Quantification of E (results of short exposure were quantified). **(G)** Diagram of working hypothesis that the synergetic effects of variations in ARHGAP31 and FBLN1 cause penetrance of terminal transverse limb defects.

As Cdc42, a key regulator of cell cycle, is a putative target of GTPase-activating activity of ARHGAP31, we particularly examined Cdc42 expression and activity in mutants-transfected cells to further understand how mutant ARHGAP31 and FBLN1 contribute to apoptosis and cytotoxicity. Total Cdc42 levels were not altered by ARHGAP31 mutation, or FBLN1 mutation, or between co-transfected groups (Figures 7A,B). Next, we performed pull-down assay to analyze the level of active Cdc42 (GTP-bound form) in the cell lysate. In agreement with previous finding that AQX truncation showed decreased Cdc42 activity, E875K mutant in our study indicated similar inhibition of Cdc42 activation. Surprisingly, FBLN1 variation showed consistent inactivation of Cdc42. When co-transfected with AM, FBLN1 mutation further decreased the amount of active Cdc42 (Figures 7C,D).

In addition, we attempted to explore other possible targets for both ARHGAP31 and FBLN1. As a result, when comparing AM + FM to AW + FW groups, ARHGAP31 and FBLN1 mutants together showed significantly elevated the activation of MAPK/ERK pathway at both 24 h and 48 h after transfection (Figures 7E,F). Interestingly, a two-fold activation in the AW + FM group was observed at 24 h, indicating that MAPK/ERK activation may be initially driven by the FM variant. Since the MAPK/ERK signaling pathway is widely involved in cell activities including cell proliferation, differentiation, and cell apoptosis or anti-apoptotic functions, we thus speculate both Cdc42 and MAPK/ERK pathways play roles in synergistic effects of impaired cell activity and accelerated cell death *in vitro*, contributing to the clinical manifestation of limb defects (Figure 7G).

## Discussion

Adams-Oliver syndrome is classically characterized by a combination of ACC and TTLDs, based on the diagnostic criteria proposed by Lehman in 2016 (Lehman et al., 1993). Six causative genes with variations have been identified in relation to AOS including *ARHGAP31*, *DOCK6*, *EOGT*, *RBPJ*, *NOTCH1*, and *DLL4*. In a four-generation family with *ARHGAP31* variation, all cases with *ARHGAP31* variations showed TTLD indicative of high penetrance, suggesting *ARHGAP31* variation may only cause isolated phenotype of TTLD in AOS (Isrie et al., 2014). Previously, all pathogenic variations in *ARHGAP31* reported are located within terminal exon 12, leading to premature termination of the translated protein or a missense mutation at C-terminus of the protein (Southgate et al., 2011; Isrie et al., 2014; Tao et al., 2021). Here in our case, the missense mutation (c.2623G > A (p.Glu875Lys)) occurs tween two proline-rich domains of the protein sequence in ARHGAP31 and is a rare variant ever been reported. The inherited copy in the daughter showed typical TTLD on here hands and feet, which are consistent with typical limb abnormalities in AOS (Isrie et al., 2014; Hassed et al., 2017). WES and Sanger sequencing excluded possibility of *de novo* variation or typical recessive inheritance of variants in single gene, therefore the transition of normal to abnormal expressivity in the family lead us to question the underlying cause. Since variation in *FBLN1* has been shown to be associated with complex forms of syndactyly (Debeer et al., 2002; Bohlega et al., 2014), the extra variation in *FBLN1* gene was therefore suspicious to be the other candidate as a causative gene. Due to the aforementioned points, we proposed that two gene variants together in the daughter may account for the dominant phenotype of limb malformations.

ARGHAP31 functions as a GAP by hydrating GTP to GDP, thereby reducing the level of GTP-bound Cdc42 and inactivating GTPase Cdc42/Rac1. Therefore, ARGHAP31 is required for cell spreading, polarized lamellipodia formation, and cell migration (Kurokawa et al., 2004; Tcherkezian et al., 2006). Southgate et al. (2011) demonstrated that truncated mutations in the last exon of *ARGHAP31* were pathogenic due to gain of function. Two such mutations, *p*. Gln683X (AQX named in our study) and *p*. Lys1087SerfsX4, led to generation of two fragments and decreased the amount of active Cdc42 levels. Here, we confirmed the toxicity of AQX using the CCK-8 assay and found that rare missense mutation E875K in ARHGAP31 showed less toxicity compared with AQX (Figure 6D). In addition, no effects on full-length ARHGAP31 expression, cleavage or cellular distribution were observed, although consistent spontaneous cleavage was observed throughout assays. To some degree, the unprevailing mutation may explain the normal phenotype in the mother as no apparent changes were observed by mutant ARHGAP31 in overexpressing cells. Labeling exogenous expression of ARHGAP31 with small FLAG tag, confirmed by anti-ARHGAP31 staining (Figure 2D), showed that the GAP protein is distributed at cytosolic prone to the plasma membrane, accompanied with nuclear localization, which was not altered by the E875K mutation. In disagreement, endogenous ARHGAP31 was reported to be localized to the Golgi (Southgate et al., 2011), and our result clearly showed that ARHGAP31 is not, least not exclusively, localized in the Golgi apparatus (Figure 2E). In addition, we believe that peri-membrane localization is more rational for the protein to function as a GAP when sensing broad extracellular signals.

Fibulins are extracellular glycoproteins secreted in elastic fibers and basement membranes of various tissues where they interact with several extracellular matrix components (De Vega et al., 2009). FBLN1-encoded Fibulin-1 is prominently expressed in the neural crest cells during development and is implicated in tissue organogenesis in developing myotomes, endocardial cushion and digits of the developing limbs (Cooley et al., 2008; De Vega et al., 2009). A translocation involving the last exon of FBLN1 isoform D has been associated with polysyndactyly possibly due to haploinsufficiency of isoform D (Debeer et al., 2002). Using a combination of homozygosity mapping and exome sequencing, the first point mutation in Fibulin-1 has also been confirmed in a novel autosomal recessive syndrome in development (Bohlega et al., 2014). In our study, HeLa cells expressing the rare variant of Fibulin-1 (R550H) showed compromised protein levels, but unaltered subcellular distribution. FBLN1-deficient embryos showed increased apoptosis in subpopulation of neural crest cells (Cooley et al., 2008). Inhibition of Fibulin-1 sensitized cancers cells to apoptotic signals, suggesting that FBLN1 serves a protective role from cell apoptosis (Gong et al., 2020). The R550H mutation-induced reduction of Fibulin-1 was moderated when co-expressed with wild-type ARHGAP31 but was consistently observed when mutant ARHGAP31 was co-transfected in both cell lines. In elucidation of the reason for reduced FBLN1 mutant expression, we found increased transcription levels for the mutant (Figures 3E,F); however, protein degradation rate was obviously faster in the mutant, suggesting the mutation-induced protein instability (Figure 3G). In conclusion, the reduction of Fibulin-1 widely observed in our study again is consistent with the indication that haploinsufficiency of Fibulin-1 may be able to initiate cell apoptosis during development.

Suggested by CCK-8 assay which indicates cell proliferation and cytotoxicity, FBLN1 mutation showed moderate cytotoxicity but has no effect on cell proliferation compared to the wild-type (Figures 6B,E,F); however, when cell viability and cell death were investigated in co-transfections, synergistic effects of two mutants were consistently observed (AM + FM vs. AW + FW). In particular, cell apoptosis was confirmed in line with the cell death data. These findings are indeed intriguing and important in supporting the synergistic effects by two mutants in both *in vitro* and in the patient. Previously, few evidence elucidating functions of ARHGAP31 in AOS or FBLN1 in synpolydactyly have been reported. Combining our preliminary data of sub-localization distribution, it is suggested that these two proteins may have inter-molecular interactions, which may be enhanced by mutant epitopes, as elevated co-localizations with mutant proteins were found. *In vivo* evidence on their interactions might be supportive. More interestingly, functional assessments by CCK-8 assay, cell proliferation, and cell apoptosis together suggest a global loss of function in group co-expressing two mutants, coincided with the penetrance of TTLD in the mutant carrier.

As a GAP, ARHGAP31 inactivates Cdc42/Rac1 by dehydrating GTP to GDP that were bound by Cdc42 (Caron et al., 2016). The truncated form of C-terminus of ARHGAP31 boosts the activity of the RhoGAP domain by specific interaction with 1–221aa fragment, leading to decreased activity of Cdc42/Rac1 (Southgate et al., 2011). Rho GTPases such as Cdc42 regulate numerous cell functions, including cell cytoskeleton organization, migration, gene transcription, adhesion, cellular proliferation, and survival (Mosaddeghzadeh and Ahmadian, 2021). Pathogenic Cdc42 variant has been recently reported in AOS and other developmental disorders (Martinelli et al., 2018; Schnabel et al., 2021). Consistently, using PAK-PBD beads, we successfully pulled down active GTP-bound Cdc42 and found its activity was decreased by ARHGAP31 mutant, FBLN1 mutant, and the two mutants together (Figures 7C,D). It is possible that single variation induced overactivation of ARHGAP31, leading to Cdc42 inactivation, which was further augmented in the presence of mutant FBLN1, leading to a further reduction of activating Cdc42. Surprisingly and intriguingly, we disclosed significant activation of MAPK/ERK presenting the AM + FM group, further supporting the synergistic effects observed by the functional analysis (Figure 7E).

In summary, our findings identified two rare heterozygous variants in AOS risk gene *ARHGAP31* and synpolydactyly risk gene *FBLN1* in one case with TTLD deformation. Heterozygous carrier of individual variant shows no phenotype whereas two variants together resulted in typical limb defects. *In vitro* assays indicate that only two mutants could synergistically exhibit decreased cell viability, impaired cell proliferation, and increased apoptosis activation, possibly triggered by Cdc42 inactivation and MAPK/ERK activation, which both mutants have effects on (Figure 7G). Taken together, our study provides evidence of a synergistic disruption of cellular functions attributed by two independent risk gene variants, expanding the clinical spectrums of causal gene interactions in hereditary TTLD and AOS etiology.

## Data Availability

The datasets presented in this study can be found in online repositories. The names of the repository/repositories and accession number(s) can be found below: https://www.ddbj.nig.ac.jp/, LC700997 https://www.ddbj.nig.ac.jp/, LC700998 https://www.ddbj.nig.ac.jp/, LC700999 https://www.ddbj.nig.ac.jp/, LC701000.
